# T2T Genomes Unveil Centromere Architecture and Adaptive Divergence in Large Yellow Croaker (*Larimichthys crocea*)

**DOI:** 10.1002/advs.202506374

**Published:** 2025-08-22

**Authors:** Yu Cui, Yingbo Yuan, Bi Wang, Baolan Wu, Mingyi Cai, Ming Fang, Weiliang Shen, Xiongfei Wu, Fang Han, Zhiyong Wang

**Affiliations:** ^1^ Key Laboratory of Healthy Mariculture for the East China Sea Ministry of Agriculture and Rural Affairs Jimei University Xiamen 361021 China; ^2^ Aquatic Technology Promotion Section Ningbo Academy of Oceanology and Fishery Ningbo Zhejiang 315000 China

**Keywords:** adaptive evolution, centromeric, Larimichthys crocea, SVs, T2T‐genome, 5S rRNA

## Abstract

The large yellow croaker (*Larimichthys crocea*) is a key aquaculture species, yet gaps in high‐quality genomes hinder studies of centromeric and adaptive evolution. This study presents two telomere‐to‐telomere (T2T), gapless genomes from the Min‐Yuedong (MYD) and Daiqu (DQ) populations, which diverged 4.18 million years ago. The centromeres are characterized by a 42 bp tandem repeat (Cen‐42) and invasions by endogenous retrovirus 1 (ERV1) of long terminal repeat (LTR) elements. Both populations exhibit transcriptional activity in centromeric regions, but display significant divergence in gene number and composition, likely driven by rapid population expansion and selective pressure. In addition, 5S ribosomal RNA genes form ultra‐large tandem repeat clusters in the short‐arm regions of more than 10 chromosomes. The T2T genome assemblies resolve previously unassembled regions, identifying 533 and 351 new genes in T2T‐MYD and T2T‐DQ, respectively. Four population‐specific structural variations, located in *pla2g4a*, *eno2*, *ptprb*, and *itrp3* are identified. Comparative genomic analyses highlight distinct adaptive features between the two populations, including differences in metabolic efficiency, arachidonic acid metabolism, chemosensasory function, and circadian rhythm. Collectively, these findings deepen the understanding of *L. crocea* evolution and provide valuable genomic resources for its conservation and breeding.

## Graphical abstract

This study presents telomere‐to‐telomere genome assemblies for two populations of *Larimichthys crocea*. We identified centromere‐specific tandem repeats invaded by LTR/ERV1 retrotransposons, unique 5S rRNA enrichment patterns, and population‐specific structural variants. Comparative genomic analyses further reveal distinct adaptive mechanisms in the MYD and DQ populations, potentially driven by their respective environmental conditions.‐.

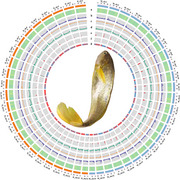

## 1 Introduction

The Sciaenidae family, encompassing species such as the large yellow croaker (*Larimichthys crocea*), red drum (*Sciaenops ocellatus*), and meagre (*Argyrosomus regius*), represents one of the most rapidly expanding and economically significant groups in global aquaculture. Among them, *L. crocea* is the most extensively cultivated, comprising over 60% of global Sciaenidae fish production, with China as the leading producer.^[^ ^1^ ^]^ Its high market demand, exceptional taste, and rich nutritional value have further driven its commercial prominence in East Asia and beyond.^[^ ^2^, ^3^ ^]^ Based on geographic distribution and morphological traits, *L. crocea* populations along the Chinese coast are categorized into three major groups: the Min‐Yuedong population (MYD), the Daiqu population (DQ), and the Naozhou population (NZ). The MYD is distributed in the southern East China Sea and the Taiwan Strait, the DQ resides in the East China Sea, and the NZ is located in the western South China Sea.^[^ ^4^ ^]^ Population genetic analyses indicate distinct adaptive landscapes between the northern cold‐region population (DQ) and the southern warm‐region populations (MYD and NZ).^[^ ^5^ ^]^ However, the molecular mechanisms underlying their adaptation to divergent environmental pressures remain unclear. Since 2014, multiple genome assemblies for the MYD population have been published,^[^ ^6^, ^7^, ^8^, ^9^, ^10^ ^]^ progressively improving in quality (Table S1, Supporting Information). However, issues like assembly gaps and uncharacterized repetitive sequences persist. Recently, chromosome‐level assemblies for the DQ and NZ populations have been reported.^[^ ^11^ ^]^ Yet they remain highly fragmented, lacking fully resolved telomeric and centromeric regions. Such limitations hinder a comprehensive understanding of genome organization and evolutionary adaptation.

Since the first X chromosome T2T assembly of the human,^[^ ^12^ ^]^ near‐complete genomes have been generated for multiple species, including rice (*Oryza sativa*),^[^ ^13^ ^]^ thale cress (*Arabidopsis thaliana*),^[^ ^14^ ^]^ and goat (*Capra hircus*).^[^ ^15^ ^]^ However, even these assemblies often contain unresolved repetitive regions, particularly in telomeres and centromeres, which comprise high‐copy tandem repeats. Centromeres play a pivotal role in chromosome segregation, ensuring genomic stability during cell division.^[^ ^16^ ^]^ In most eukaryotes, centromeres are defined by species‐specific tandem repeats.^[^ ^17^, ^18^, ^19^ ^]^ To date, fully resolved telomere and centromere assemblies have been achieved in human,^[^ ^20^ ^]^ maize (*Zea mays*),^[^ ^21^ ^]^ rice (*O. sativa*),^[^ ^22^ ^]^ chicken (*Gallus gallus domesticus*)^[^ ^23^ ^]^ and sheep (*Ovis aries*).^[^ ^24^ ^]^

In fish species, reports of T2T genome assemblies including gapless versions are becoming increasingly common.^[^ ^25^, ^26^, ^27^, ^28^, ^29^ ^]^ With continuous advances in sequencing technologies and assembly strategies. The generation of gapless T2T genomes is rapidly emerging as a transformative trend in genomics. So far, research on centromeres in fish has been limited, despite the significant interspecies diversity that has been observed. For instance, the zebrafish (*Danio rerio*) centromere is predominantly composed of A/T‐rich tandem repeats,^[^ ^30^ ^]^ whereas a 524 bp repeat has been identified as the centromeric satellite DNA in the zig‐zag eel (*Mastacembelus armatus*).^[^ ^27^ ^]^ Highlighting the evolutionary plasticity of centromeric sequences in teleost genomes.

In this study, we utilized PacBio HiFi, Hi‐C, and ultra‐long Oxford Nanopore sequencing to generate the first fully complete T2T genome assemblies for two geographically distinct *L. crocea* populations. These assemblies achieve unprecedented continuity and completeness for this species, allowing precise characterization of telomeric and centromeric structures at the sequence, epigenetic, and transcriptional levels. By integrating comparative genomic and transcriptomic analyses, we explored the molecular mechanisms underlying environmental adaptation across populations. Our findings not only provide a valuable genomic resource for evolutionary and functional studies but also offer key insights into the genetic improvement and selective breeding of this economically important aquaculture species.

## 2 Results

### 2.1 T2T Genome Assembly and Annotation

We utilized PacBio HiFi, ultra‐long ONT, and Hi‐C sequencing technologies to assemble the MYD and DQ genomes. The MYD population generated 194.35 Gb of clean data, and the DQ population produced 190.35 Gb (Table S2, Supporting Information). The initial HifiASM (v0.19.8)^[^ ^31^ ^]^ assembly for MYD resulted in a 725.75 Mb genome with 79 contigs and a contig N50 of 31.92 Mb. Based on this high‐quality contiguous version, we performed chromosome‐level scaffolding using 3D‐DNA (v180922),^[^ ^32^ ^]^ resulting in an assembly size of 725.75 Mb. Although both the continuity and overall assembly quality were enhanced, several gaps persisted, and some telomeric regions remained incomplete. To overcome these limitations, Verkko (v1.4.1)^[^ ^33^ ^]^ generated a diploid genome assembly that captures the complete information of both haplotypes. This assemble consists of 824 contigs with a contig N50 of 30.28 Mb. After gap filling and refinement, the final T2T genome (T2T‐MYD) was 708.23 Mb in size, comprising 24 chromosomes, with a scaffold N50 of 31.71 Mb and a GC content of 41.57% (**Table** **1**). For the DQ population, an initial 722.30 Mb genome was assembled using HifiASM (v0.19.8),^[^ ^31^ ^]^ resulting in 101 contigs and a contig N50 of 31.87 Mb. Following scaffolding with 3D‐DNA (v180922),^[^ ^32^ ^]^ the assembly yielded a 722.50 Mb genome consisting of 98 scaffolds. Subsequently, Verkko (v1.4.1)^[^ ^33^ ^]^ generated a 1.43 Gb draft genome containing 693 contigs with a contig N50 of 30.21 Mb. After further optimization, the final T2T genome (T2T‐DQ) was 706.62 Mb, also comprising 24 chromosomes, with a scaffold N50 of 31.56 Mb and a GC content of 41.58% (**Table** **2**).

**Table 1:** Summary of assemblies of MYD.

Statistics	HifiASM	3D‐DNA	Verkko	T2T‐MYD
Number of contigs	79	78	824	24
Max length [bp]	36 800 019	36 800 019	36 797 197	36 452 242
Mean length [bp]	9 186 702	9 304 487.50	1 743 967	29 509 573.30
Contig/ScaffoldN50 [bp]	31 918 678	31 918 678	30 283 531	31 707 399
Contig/ScaffoldN90 [bp]	24 700 977	24 700 977	21 647 016	25 193 929
Total size of assembled genomes [bp]	725 749 522	725 750 022	1 437 028 801	708 229 758
Number of scaffolds	79	24	49	24
Number of telomeres [pairs]	32 (10)	36 (14)	68 (26)	48 (24)
Number of centromeres	NA	NA	NA	24
GC content [%]	49.18	49.18	57.78	41.57

**Table 2:** Summary of assemblies of DQ.

Statistics	HifiASM	3D‐DNA	Verkko	T2T‐DQ
Number of contigs	101	98	693	24
Max length [bp]	36 896 119	36 896 119	38 526 242	36 576 956
Mean length [bp]	7 151 515	7 372 412	2 065 287	29 442 335
Contig/ScaffoldN50 [bp]	31 871 358	31 871 358	30 206 031	31 563 290
Contig/ScaffoldN90 [bp]	22 466 682	24 577 710	17 422 027	24 877 144
Total size of assembled genomes [bp]	722 303 058	722 496 434	1 431 244 497	706 616 053
Number of scaffolds	101	24	50	24
Number of telomeres [pairs]	29 (10)	33 (12)	50 (15)	48 (24)
Number of centromeres	NA	NA	NA	24
GC content [%]	48.44	48.65	52.13	41.58

**Figure 1:**
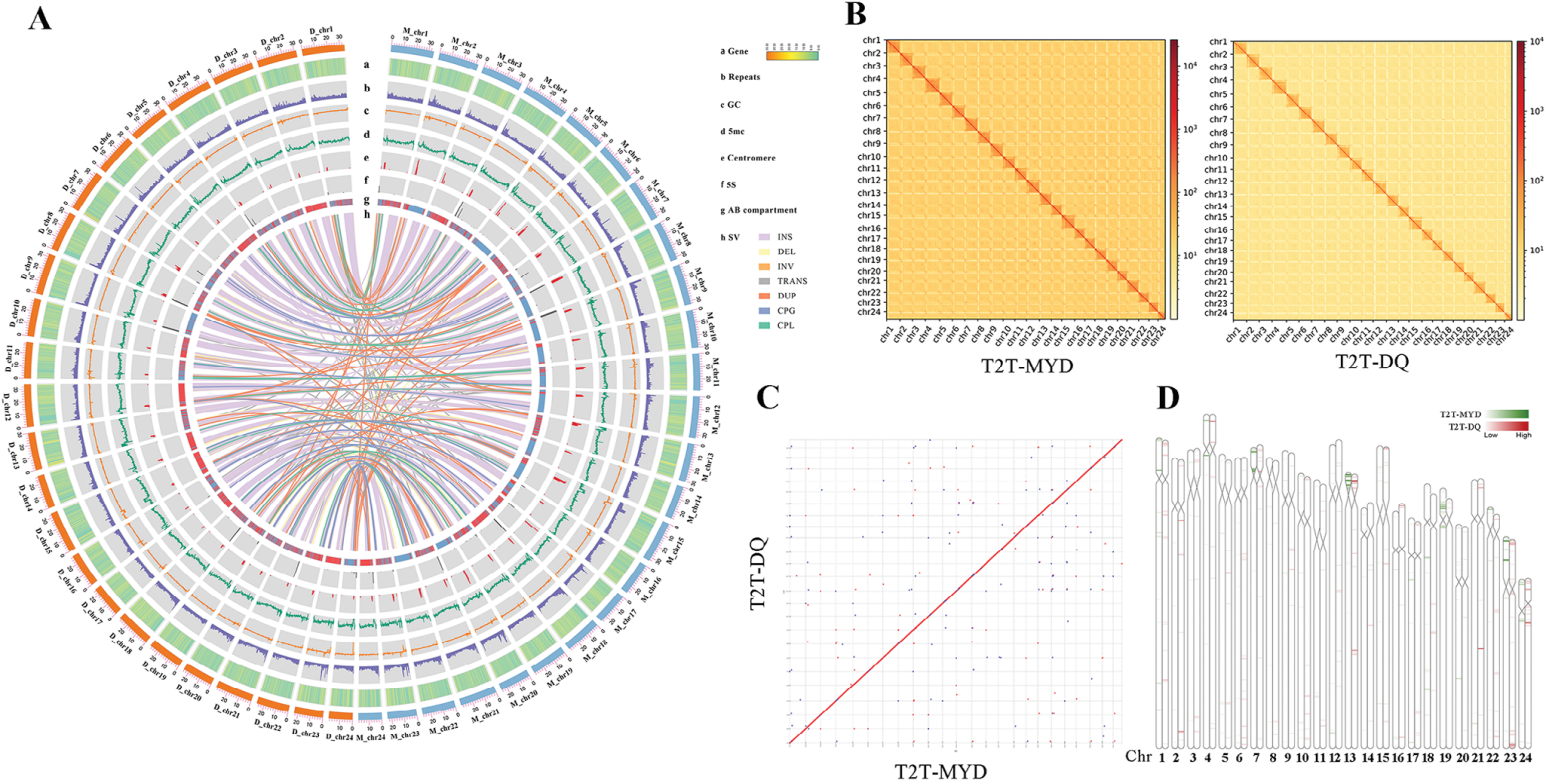
T2T assembly of two stocks of *Larimichthys crocea*. A) Genome overview of the 24 chromosomes of T2T‐MYD and T2T‐DQ (the T2T‐MYD chromosomes are in blue and T2T‐DQ chromosomes are in orange). a) Gene density. b) Density of repeats. c) GC content. d) DNA methylation distribution (5‐methylcytosine). e) Positions of centromeres along the chromosomes. f) Distribution of 5S rRNA. g) A/B compartment of the chromosome. h) SVs of two stocks. Depictions of insertions (INS), deletions (DEL), inversions (INV), translocations (TRANS), duplications (DUP), copy number gains (CPG), and complex rearrangements (CPL). All densities are calculated in 100 kb windows, from orange to green colors in density plots are for lower to higher values respectively. B) Contact maps for chromosome interaction. The heatmaps depict Hi‐C interaction matrices for T2T‐MYD (left) and T2T‐DQ (right) genomes. C) The genomic synteny between the T2T‐MYD and T2T‐DQ. It shows that there are very few SVs between the two stocks. D) Genomic locations of newly annotated genes mapped to existing genomes. Heatmaps indicate gene abundance, with green representing T2T‐MYD and red representing T2T‐DQ, darker colors denote higher gene counts.

Hi‐C contact maps confirmed successful chromosome anchoring in both genomes (**Figure** **1**B). Synteny analysis performed with MUMmer4 (v4.0.0)^[^ ^34^ ^]^ revealed high genomic consistency between populations (Figure 1C). Mapping of Illumina, HiFi, and ONT reads to the T2T genomes resulted in 99.92% coverage across all datasets (Table S3, Supporting Information), confirming sequencing completeness and consistency. Genome quality evaluation using Merqury (v1.3)^[^ ^35^ ^]^ produced QV scores of 52.89 for T2T‐MYD and 53.07 for T2T‐DQ. Completeness assessed by BUSCO^[^ ^36^ ^]^ and Compleasm^[^ ^37^ ^]^ was 99.3% and 99.97% for T2T‐MYD, 99.2% and 99.98% for T2T‐DQ respectively. Genome compactness index (GCI)^[^ ^38^ ^]^ scores were 41.54 and 40.84, further supporting the high‐quality nature of the assemblies (Table S4, Supporting Information).

Repetitive sequences constituted 29.32% (207.63 Mb) and 28.79% (203.46 Mb) of the T2T‐MYD and T2T‐DQ genomes, respectively. Four types of transposable elements (LTR, LINE, DNA, and RC) were identified, with LTR elements predominating in both genomes (38.85% in MYD versus 41.48% in DQ; Figure S1A, Table S5, Supporting Information).

A total of 26 144 protein‐coding genes (71 476 transcripts) in T2T‐MYD and 26 949 genes (82 761 transcripts) in T2T‐DQ were annotated using an integrated pipeline combining de novo prediction and homology‐based methods. Functional annotation rates showed high concordance between two genomes: NR (>97%), TrEMBL (>96%), Swiss‐Prot (>84%), Pfam (>82%), KOG (≈65%), KEGG (≈59%), GO (≈54%), and COG (≈21%; Table S6, Supporting Information). Annotation of non‐coding RNAs identified 17 189 rRNAs and 6630 tRNAs in T2T‐MYD versus 14 781 rRNAs and 6602 tRNAs in T2T‐DQ (Table S7, Supporting Information). BUSCO analysis confirmed 98.7% completeness of conserved genes in both assemblies (Table S4, Supporting Information).

Comparative genomic analysis identified 533 and 351 novel genes in T2T‐MYD and T2T‐DQ, predominantly located in centromeric and telomeric regions that have been historically challenging to assemble (Figure 1D, Table S8, Data S1, Supporting Information). These findings demonstrate that the T2T assemblies produced in this study represent a substantial advancement in resolving the complex repeat regions in the *L. crocea* genome.

### 2.2 Genomic Characterization of Telomeres, Centromeres, and 5S rRNA in *L. crocea*

**Figure 2:**
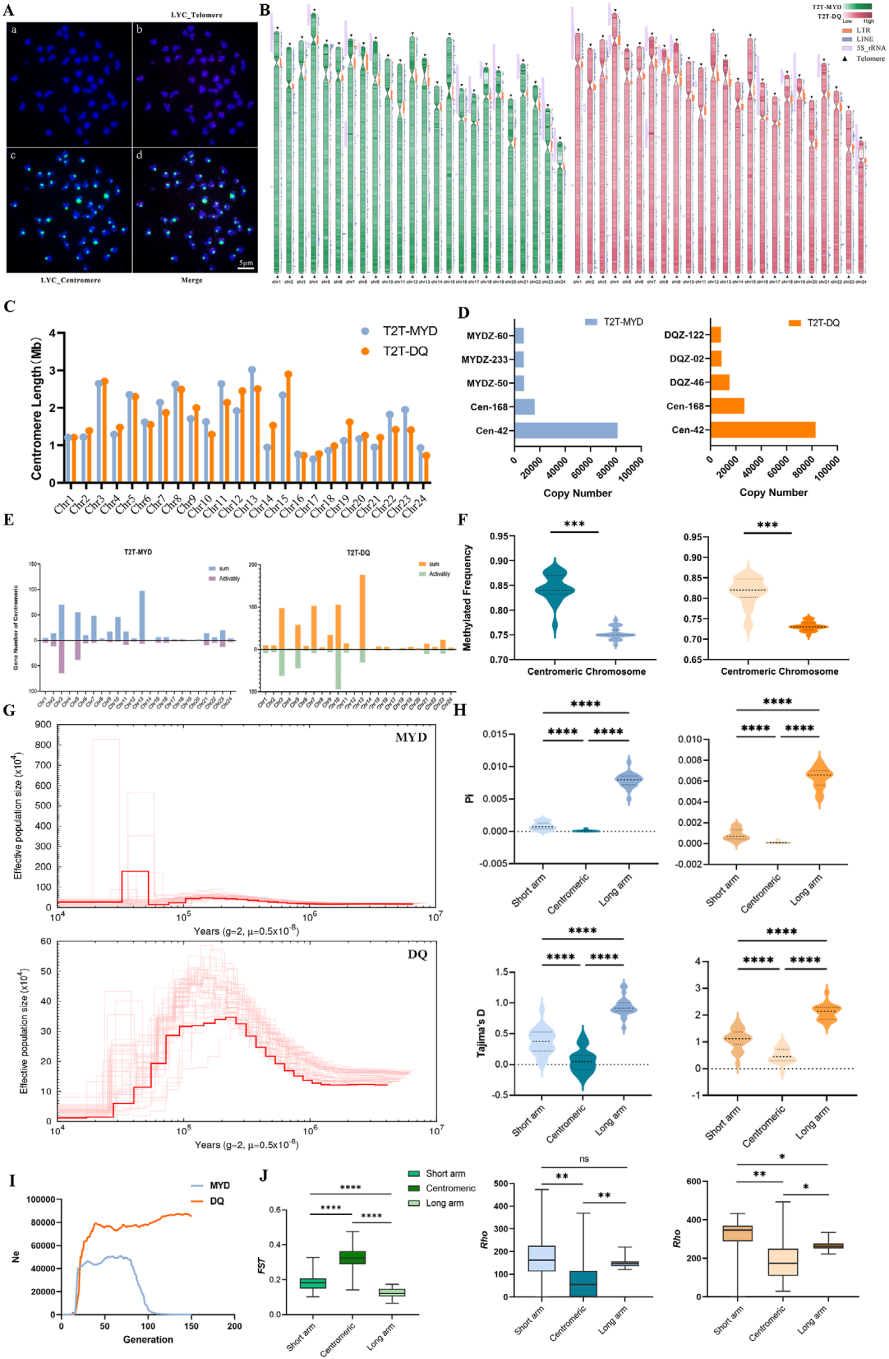
Characteristics of centromeres and telomeres in *Larimichthys crocea*. A) FISH images for probes of “TTAGGG”*6 (telomere, red) and the Cen‐42 (centromere, green) on *L. crocea* metaphase chromosomes. B) Genomic distribution patterns of centromeres, telomeres, LTRs, LINEs, and 5S rRNA elements in T2T‐MYD (left) and T2T‐DQ (right) assemblies. Green and red heatmaps indicate DNA methylation frequencies; orange bars represent LTRs, blue bars for LINEs, purple bars for 5S rRNA, and black triangles mark telomere positions. C) The centromere length on each chromosome was measured in megabases (Mb). D) Top 5 most abundant repeat elements in terms of copy number across the whole genome. Blue bars represent T2T‐MYD, and orange bars represent T2T‐DQ. E) The number of genes located within the centromeric regions of each chromosome is shown, with the upper portion representing the total number of genes and the lower portion indicating the number of actively expressed genes. The left panel corresponds to the T2T‐MYD, while the right panel corresponds to the T2T‐DQ. F) DNA methylation frequency within centromeric regions and across entire chromosomes. Left panel: T2T‐MYD; right panel: T2T‐DQ. G) The effective population size estimates for the two populations use PSMC: the upper section represents the MYD population, while the lower section represents the DQ. The *g* represents the time required to reproduce one generation, and the u means the mutation rate. H) *π*, Tajima's *D* and *ρ*‐values calculated for short‐arm, centromeric, and long‐arm regions using 20 individuals of MYD (left) and 21 individuals of DQ (right). I) The effective population size estimates for the two populations use GONE2: blue means MYDZ and orange means DQZ. J) FST calculated for short‐arm, centromeric, and long‐arm regions in T2T‐MYD.

Canonical telomeric repeats “TTAGGG/CCCTAA” were detected at both termini of all 24 chromosomes in both T2T‐MYD and T2T‐DQ assemblies (**Figure** **2**B), with average copy numbers ranging from 1295 to 1352 per chromosomal end (Figure S1D,E, Table S9, Supporting Information).

For centromeres, analysis revealed the presence of two dominant repeat sequences across all chromosomes. Among the top five repeats with the highest copy numbers, a 42 bp repeat (Cen‐42) and a 168 bp repeat (Cen‐168) were identified, with the latter comprising four tandem Cen‐42 units, two of which contained base variations (Figure 2D, Table S10, Supporting Information), forming a complex higher‐order repeat structure. In the T2T‐MYD population, the average centromere length was 1.78 Mb, with chromosome 13 being the longest (3.02 Mb) and chromosome 17 the shortest (0.63 Mb). The long arm/short arm (L/S) ratio ranged from 4 to 36, classifying 13 chromosomes as submetacentric and 11 as subtelocentric. In contrast, the T2T‐DQ population exhibited an average centromere length of 1.66 Mb, with chromosome 15 being the longest (2.9 Mb) and chromosomes 16 and 24 being the shortest (0.73 Mb; Figure 2C, Table S11, Supporting Information). The L/S ratio ranged from 4 to 24, resulting in 12 chromosomes classified as submetacentric and 12 as subtelocentric (Figure 2B, Table S11, Supporting Information). Fluorescence in situ hybridization (FISH) experiments further confirmed the chromosomal localization of Cen‐42 repeats and telomeric (TTAGGG)_n_ probes, corroborating the genome assembly results (Figure 2A). To investigate the conservation of centromeric repeats in Sciaenidae, we performed a comparative analysis of Cen‐42 distribution patterns across three species: *Larimichthys polyactis*, *Nibea albiflora*, and *Collichthys lucidus*. The Cen‐42 was ubiquitously present on all 24 homologous chromosomes in all three species. However, statistical analysis revealed interspecific variation in Cen‐42 copy numbers: *L. polyactis* exhibited the highest abundance (*n* = 95 405, pident = 97.33%, *e*‐value = 1.15e^−9^), even surpassing *L. crocea* (*n* = 81488/82977). In contrast, markedly lower copy numbers were observed in *N. albiflora* (*n* = 12 879, pident = 97.10%, *e*‐value = 6.17e^−10^) and *C. lucidus* (*n* = 24 879, pident = 96.34%, *e*‐value = 1.33e^−9^; Figure S1C, Table S12, Supporting Information).

We also observed that the centromeric regions in *L. crocea* were highly enriched with transposable elements (TEs), particularly long terminal repeats (LTRs; Figure 2B). The T2T‐MYD and T2T‐DQ genomes contained 46 280 and 49 296 copies of LTRs exceeding 100 bp, respectively, with the majority classified as LTR/ERV1 (42 092 and 45 447 copies, respectively). Notably, more than 84.73% of these LTR/ERV1 elements were localized within centromeric regions (Figure S1B, Table S5, Supporting Information). In parallel, we analyzed genome‐wide 5‐methylcytosine (5mC) distribution and constructed a methylation map. The results showed that the average methylated frequency within centromeric regions was significantly higher than that of the entire chromosomes (*p* < 0.001; Figure 2F, Table S11, Data S2,S3, Supporting Information).

In *L. crocea*, the centromeric regions remain insufficiently characterized in terms of protein‐coding gene composition, transcription, and epigenetic regulation. To further characterize the genomic features of centromeric regions, we analyzed whole‐genome sequencing (WGS) and RNA‐seq data for the two populations. WGS data from 20 individuals of the MYD and 21 individuals of the DQ population were independently mapped to the T2T reference genome for high‐confidence SNP identification. Subsequently, key population genetic metrics, including nucleotide diversity (*π*), Tajima's D, and recombination rate (*ρ*) were calculated separately for each population. The results revealed a distinct gradient‐like distribution of nucleotide diversity and Tajima's D along the chromosomes. Specifically, the centromeric region exhibited substantially lower levels of nucleotide diversity compared to the short‐arm region, which itself showed reduced diversity relative to the long arms. Tajima's D values followed a comparable gradient, although their distribution varied between populations, while the recombination rate displayed a distinct trend: centromere < long‐arm < short‐arm (Figure 2H, Table S11, Supporting Information). Notably, in the MYD population, centromeric regions on more than 10 chromosomes exhibit negative Tajima's D values, indicative of potential recent selective sweeps or episodes of population expansion. To explore the underlying cause of this pattern, we inferred historical changes in effective population size for both populations. PSMC (v0.6.5)^[^ ^39^ ^]^ analysis revealed that the MYD population underwent a rapid demographic expansion ≈50 000 years ago, followed by a marked population contraction. In contrast, the DQ population experienced a more gradual expansion beginning ≈10^5^ years ago, followed by a slow decline and eventual stabilization (Figure 2G). The effective population sizes over the past 150 years were estimated using GONE2.^[^ ^40^ ^]^ In the MYD population, the analysis revealed a gradual expansion beginning ≈118 years ago, peaking ≈62 years ago, and followed by a slow decline. A pronounced contraction occurred ≈16 years ago, with Ne sharply decreasing to 226.384 ≈13 years ago. In contrast, the DQ population experienced a steady decline in effective population size throughout the past 150 years, which accelerated ≈18 years ago and culminated in a sharp drop to 123.896 ≈12 years ago (Figure 2I, Table S23, Supporting Information). These divergent demographic histories may help explain the contrasting evolutionary dynamics observed in the centromeric regions of *L. crocea*.

All RNA‐seq reads were mapped to the T2T reference genome, and population‐specific gene expression matrices were constructed. In the centromeric region of the MYD population, a total of 451 protein‐coding genes were identified, of which 207 exhibited transcriptional activity. In contrast, 696 protein‐coding genes were detected in centromeric regions of the DQ population, with 322 showing evidence of active expression (Figure 2E, Table S11, Supporting Information). To rule out the possibility that the observed difference in gene number arose from discrepancies in genome assembly quality, we further examined two additional high‐quality T2T genomes: T2T‐XX (MYD) and T2T‐YY (DQ) Statistical analysis revealed the presence of 396 genes in the centromeric region of T2T‐XX and 643 genes in T2T‐YY (Table S11, Supporting Information), thereby supporting a genuine population level difference in centromeric gene content. Notably, only 208 genes were found to be homologous between the two populations’ centromeric regions, corresponding to 162 orthogroups (Table S14, Supporting Information). This limited homology underscores a substantial degree of gene content divergence within centromeric regions across populations.

**Figure 3:**
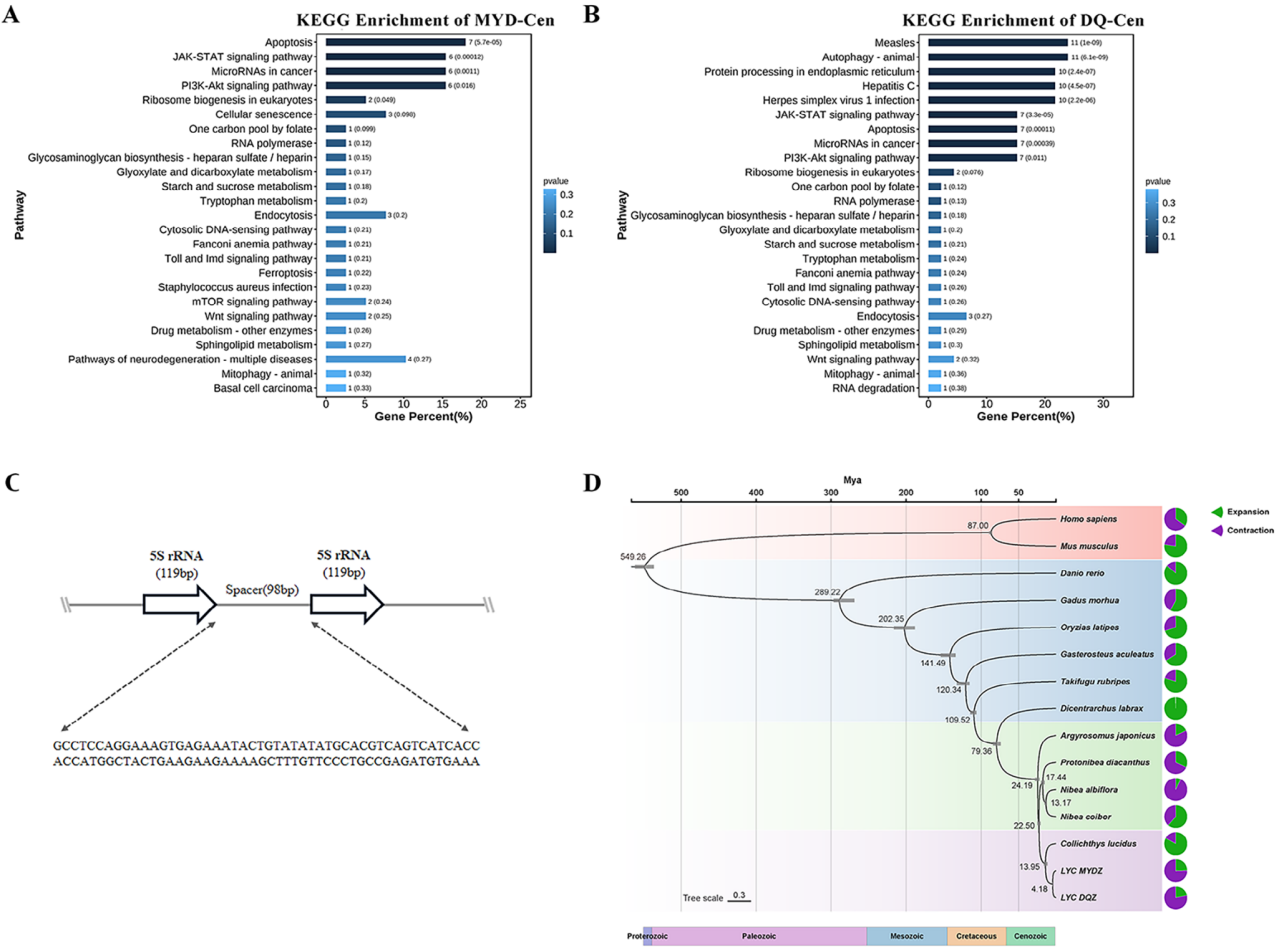
A) KEGG pathway enrichment analysis of actively expressed protein‐coding genes in the centromere of T2T‐MYD. B) KEGG pathway enrichment analysis of actively expressed protein‐coding genes in the centromere of T2T‐DQ. C) Tandem repeat structure of 5S rRNA gene units. D) Phylogenetic tree of 15 species. Phylogenetic tree of 15 representative species. The number of expanded (green) and contracted (purple) gene families is indicated along each branch.

**Figure 4:**
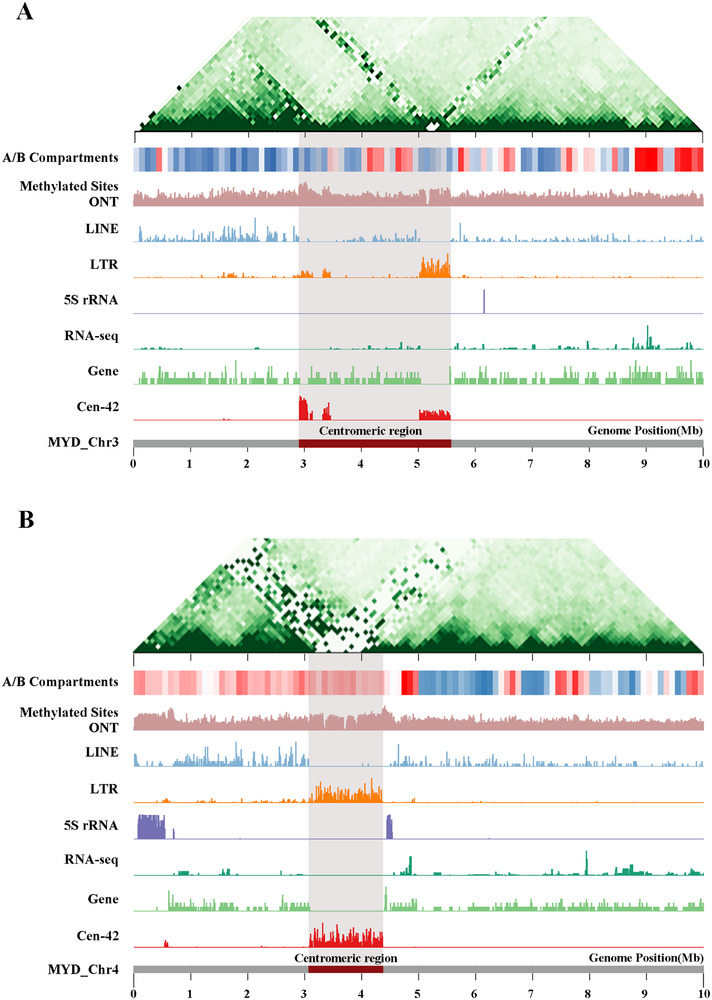
A) Genomic features of an active centromeric region (T2T‐MYD_Chr3). Shown from bottom to top: Cen‐42 density (10 kb windows); gene number; gene expression levels; 5S rRNA density; LTR density; LINE density; DNA methylation sites; A/B chromatin compartments (100 kb resolution) distribution (250 kb resolution). B) Genomic features of an inactive centromeric region (T2T‐MYD_Chr4). Shown from bottom to top: Cen‐42 density (10 kb windows); gene number; gene expression levels; 5S rRNA density; LTR density; LINE density; DNA methylation sites; A/B chromatin compartments (100 kb resolution).

To further validate the observed centromeric divergence, WGS data from both populations were aligned to the T2T‐MYD reference genome to calculate genetic differentiation (FST). The centromeric region exhibited the highest level of genetic differentiation (FST = 0.33 ± 0.07), followed by the short‐arm (FST = 0.19 ± 0.05), whereas the long‐arm displayed a moderate degree of differentiation (FST = 0.13 ± 0.03; Figure 2J, Table S11, Supporting Information). KEGG enrichment analysis of transcriptionally active genes revealed that many genes lacking detectable expression were functionally associated with viral capsid assembly and genetic material processing. In the MYD population, enriched pathways were primarily related to apoptosis, the JAK‐STAT signaling pathway, the PI3K‐Akt signaling pathway, and ribosome biogenesis in eukaryotes. In contrast, the DQ population showed enrichment not only in these pathways but also in additional immune‐related pathways, including autophagy, measles, hepatitis C, and herpes simplex virus 1 infection (**Figure** **3**AB, Table S15, Data S2–S5, Supporting Information). Notably, the centromeres of *L. crocea* exhibit extensive invasion by LTRs, and an elevated frequency of 5mC methylation. Population genetic analyses further reveal several notable characteristics of these regions, including suppressed recombination, reduced nucleotide diversity, high genetic differentiation, and the presence of transcriptionally active genes (**Figure** **4**).

Beyond the centromeric regions, the genomic distribution and evolutionary dynamics of 5S rRNA genes were also systematically investigated. In the T2T‐MYD population, 5S rRNA genes were distributed across 12 chromosomes, with more than 100 copies detected on 10 of them. Similarly, in the T2T‐DQ population, 5S rRNA genes were located on 10 chromosomes, with 8 chromosomes harboring over 100 copies (Figure 2B, Table S16, Supporting Information). This pattern is consistent with previous FISH results obtained in our laboratory.^[^ ^41^ ^]^ To determine whether this distribution pattern is unique to *L. crocea* or shared among other teleosts, we analyzed 12 recently published high‐quality genomes,^[^ ^27^, ^42^, ^43^, ^44^, ^45^, ^46^, ^47^, ^48^, ^49^, ^50^, ^51^ ^]^ focusing on the distribution characteristics of 5S rRNA genes. In most species examined, 5S rRNA genes are typically organized into tightly clustered loci, *L. crocea* displays a remarkably dispersed distribution, suggesting the occurrence of a lineage‐specific genomic rearrangement event (Table S16, Supporting Information). Notably, 5S rRNA genes in *L. crocea* are predominantly located on the short arms of chromosomes (Figure 2B) and exhibit a highly conserved 98 bp repeat interval between adjacent gene copies (Figure 3C). Furthermore, analysis of TEs content in regions containing 5S rRNA genes revealed a striking co‐localization with the LINE/L2 retrotransposon family. This co‐localization pattern is conserved across both the MYD population (with 12 chromosomes) and the DQ population (with 10 chromosomes; Table S17, Supporting Information).

### 2.3 Adaptive Evolutionary Responses of the Two Populations to Environmental Variation

**Figure 5:**
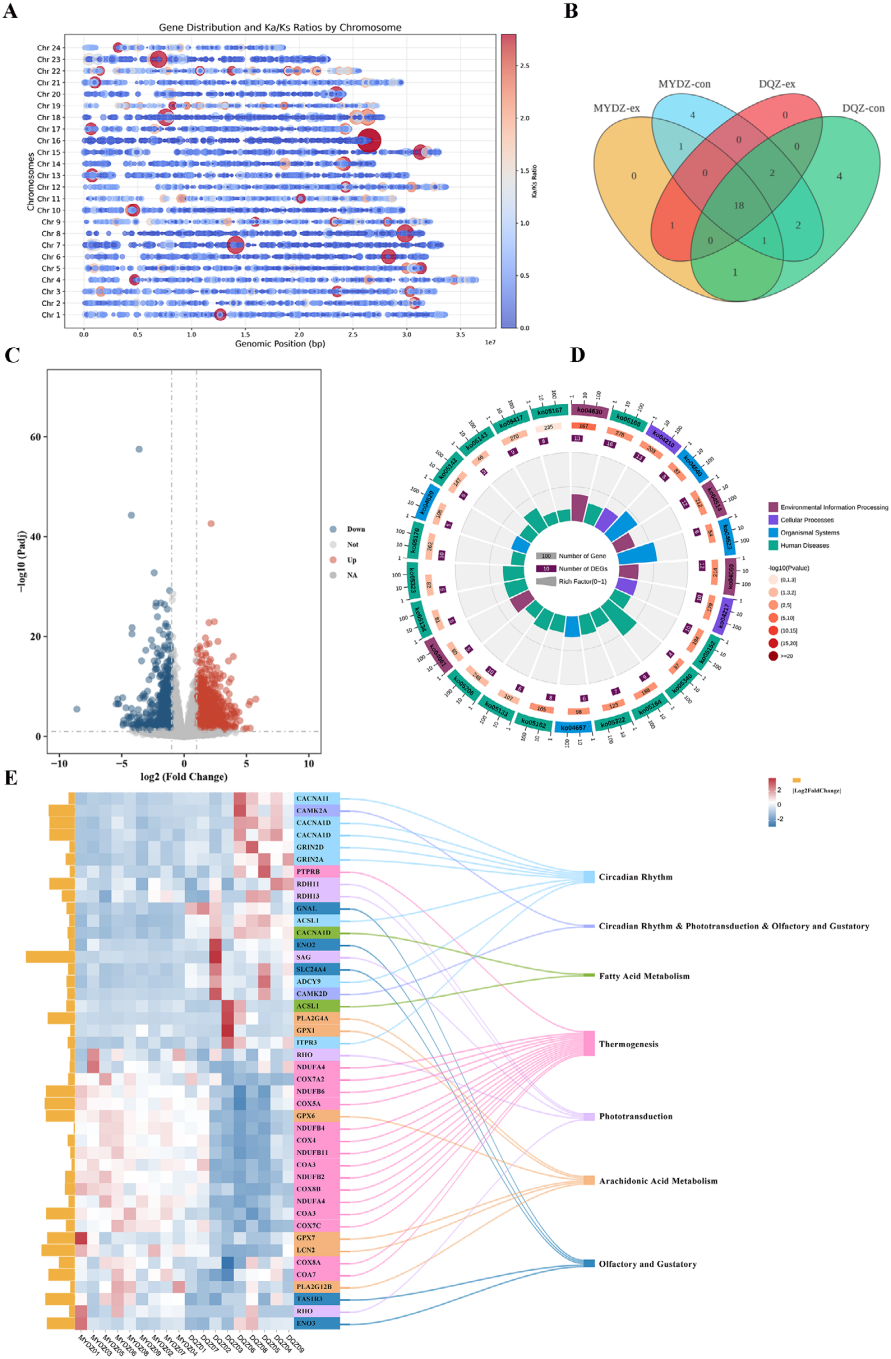
A) Genomic distribution of Ka/Ks, with dot size representing the Ka/Ks ratio. B) Venn diagram of enriched pathways from expanded and contracted gene families in T2T‐MYD and T2T‐DQ. C) Volcano plot of differentially expressed genes (DEGs), with blue indicating downregulated genes and red indicating upregulated genes. D) KEGG enrichment analysis of positively selected genes. E) Heatmap of DEGs in brain tissue between two *Larimichthys crocea* populations. The heatmap displays the expression profiles of DEGs in the brain tissue of MYD and DQ groups. Yellow bars indicate fold changes in gene expression. Functional annotation of DEGs is color‐coded: blue boxes represent genes involved in circadian rhythm; pink boxes indicate genes enriched in thermogenesis pathways; fluorescent green denotes vision‐related genes; beige represents genes related to olfaction and gustation; light green indicates genes associated with fatty acid metabolism; magenta corresponds to genes involved in arachidonic acid metabolism; and red marks genes that participate simultaneously in circadian rhythm, vision, olfaction, and gustation functions.

Due to the limited genetic resources, the evolutionary divergence mechanisms of these populations remain underexplored. The successful T2T genomes now provide a crucial foundation for in‐depth comparative genomic analyses. Divergence time estimation suggests that MYD and DQ populations diverged ≈4.18 million years ago (Figure 3D). Gene family analysis in T2T‐MYD genome identified 26 144 genes, of which 25 592 were assigned to 17 807 gene families, including 25 MYD‐specific families comprising 169 genes. In comparison, the T2T‐DQ genome contains 26 949 genes, with 26 327 genes classified into 17 840 families, including 58 DQ‐specific families encompassing 588 genes. A total of 21 972 orthologous genes were shared between the two populations, corresponding to 17024 orthogroups. Gene family evolution analysis revealed 45 expanded and 139 contracted gene families in T2T‐MYD, and 46 expanded and 164 contracted families in T2T‐DQ (Figure 3D, Table S18, Supporting Information). Selection pressure analysis identified 1062 collinear blocks, with the largest located on chromosome 18 (1141 genes) and the smallest on chromosome 24 (483 genes; Data S10, Supporting Information). Ka/Ks analysis of 19 405 orthologous gene pairs revealed 913 genes under positive selected in the DQ genome (**Figure** **5**A, Data S11, Supporting Information).

To further investigate the differences between the two populations, we conducted functional enrichment analysis of the expanded and contracted gene families. In the MYD population, expanded gene families were significantly enriched in immune‐related pathways, including the Toll and Imd signaling, NOD‐like receptor signaling, and cytokine–cytokine receptor interaction pathways (Figure S2A, Data S6, Supporting Information). Notable expansions were also observed in fatty acid metabolism pathways, particularly those involving the *elovl1* and *aloxe3* genes, which encode key enzymes in fatty acid elongation and arachidonic acid biosynthesis. In contrast, the *acsl1* gene, associated with fatty acid biosynthesis, was contracted in MYD (Figure S2A, Table S19, Supporting Information). In the DQ population, we observed expansion of genes related to anaerobic metabolism, such as *eno1*, *eno2*, *eno3*, and *pik3ca*. Gene families involved in phototransduction and retinol metabolism (e.g.,* rdha11* and *rdh13*) were contracted, while those associated with olfactory and gustatory functions, such as *tas1r1*, were expanded. Interestingly, while the *elovl1* gene family was expanded in MYD, it was contracted in the DQ population (Figure 5B; Figure S2B, Tables S15,S19, Data S8,S9, Supporting Information).

A total of 913 positively selected genes were enriched in the pathways mentioned above (Table S15, Data S12, Supporting Information). Specifically, *cyp3a4* and *pla2g4a* were enriched in the arachidonic acid metabolism, while *ptprb, slc25a29, atp5pb* and COX family were enriched in the thermogenesis pathway. Several genes associated with olfactory and gustatory transduction—including *calm1, pde1a, or4n2, or5f1, or52b2, kcnk5, and p2ry1*—also exhibited signatures of positive selection (Figure 5D, Table S19, Data S12, Supporting Information).

By integrating gene family evolution and positive selection analysis, we identified key differences in gene sets associated with adaptive traits, such as metabolic efficiency, fatty acid metabolism, phototransduction, and chemosensation. We further focused on 87 candidate adaptive genes that exhibit significant expansion/contraction (*p* < 0.05) or positive selection signals. Structural variation (SV) analysis based on high‐depth WGS data revealed SVs associated with these genes, and RNA‐seq data were used to assess their expression profiles. This multidimensional framework enabled us to investigate the coordinated evolution of adaptive genes at both the molecular and transcriptional levels.

SV detection was performed using HiFi reads from three MYD individuals and two DQZ individuals. A total of 138 823 SVs were identified, consisting primarily of deletions (DEL, 50.23%) and insertions (INS, 48.01%), with a length peak between 50 and 100 bp (Figure 1A, Figure S1E,F, Supporting Information). Among the 87 candidate genes, 40 SVs were detected, including 13 DELs and 27 INSs. These SVs were distributed as follows: three in the 5′ UTR, five in the 3′ UTR, and 32 within introns. Notably, five SVs were detected in the *ITPR3* gene, including one in the promoter and three in the first intron (Table S20, Supporting Information). To validate population specificity, we aligned the high‐depth WGS data to the T2T‐DQ genome and filtered for SVs exclusive to the MYD population. Ultimately, four population‐specific SVs were identified in the *pla2g4a*, *eno2*, *ptprb*, and *itpr3* genes. These variants are functionally enriched in the arachidonic acid metabolism, olfactory and gustatory, thermogenesis, and circadian rhythm pathways, respectively. Genotyping analysis of the *ptprb* variant (chr5_31503914) revealed a consistent heterozygous 0|1 genotype in all 20 MYD individuals (Table S21, Supporting Information).

To explore potential differences in the expression levels of the 87 candidate genes, we performed transcriptome sequencing on brain tissue samples from 9 individuals with MYD and 9 individuals with DQ population. A total of 897 genes were significantly upregulated and 504 were downregulated in the MYD population (|log2FoldChange| > 1 and *P*adj ≤ 0.05; Figure 5C, Table S22, Data S13, Supporting Information). Among the 87 candidate genes, 23 were significantly downregulated and 34 were significantly upregulated, 12 showed no significant expression change, and 22 genes were not detected (Figure 5E, Table S19, Supporting Information). Among the 17 genes enriched in fatty acid metabolism, one copy of *elovl1* was significantly upregulated. Of the four copies of *acsl1*, one was downregulated and two were upregulated. In the arachidonic acid metabolism pathway, all genes were significantly upregulated except for *pla2g4a*, which contains a 299 bp deletion in the 3′ ULR. Among the 20 genes related to metabolic efficiency, all were upregulated except *slc25a29* (downregulated) and *cox6a1* (no significant change). Notably, *ptprb* which carries an SV, was also upregulated. For genes involved in circadian rhythm, *notch1* and *myl6* were significantly upregulated, whereas the remaining 10 genes were downregulated –including *itpr3* (with SV) –and all three copies of *cacna1d* were strongly downregulated, with the largest fold change reaching 10.64. In vision‐related pathways, *rdh* and *rho* were upregulated, while *sag* and *camk2* were downregulated. Among the 28 genes enriched in the olfactory and gustatory pathways, both copies of *eno3* and *tas1r3* were upregulated, whereas *pik3ca*, *tas1r1*, *kcnk5*, *slc24a4*, *camk2a*, *gnal*, and *camk2d* were downregulated. Interestingly, although a 56 bp INS was detected in *eno2*, no significant difference in its transcriptional level was observed (Figure 5E; Figure S2C, Tables S15,S19, Data S14,S15, Supporting Information).

## 3 Discussion

In this study, we systematically elucidated the centromeric sequence characteristics and adaptive evolution mechanisms of the large yellow croaker by constructing high‐quality T2T genomes for two geographical distinct populations. Unlike traditional genome assemblies, the T2T genomes enabled the resolution of highly repetitive regions, such as centromeres, providing novel insights into the relationship between genome structural evolution and ecological adaptation.

We identified a Cen‐42 tandem repeat motif in the centromeric region of *L. crocea*. This motif is conserved across other Sciaenidae species, including *L. polyactis*, *N. albiflora*, and *C. lucidus*, suggesting a critical role in centromere function. The lower copy numbers of Cen‐42 in *N. albiflora* and *C. lucidus* are likely attributable to differences in genome assembly strategies, particularly the lack of ONT reads. This underscores the necessity of long‐read sequencing technologies for accurate detection of repetitive elements. BLASTN comparison revealed over 96% sequence identity, indicating strong evolutionary conservation, though functional mechanisms remain to be clarified. Centromeres are known to evolve rapidly, driven by high turnover rates of tandem repeats and transposable elements.^[^ ^52^ ^]^ This pattern is evident across species: human centromeres are rich in LINEs and SINEs;^[^ ^19^ ^]^ *Arabidopsis thaliana* with Ty1/Copia elements in CENH3‐binding regions;^[^ ^53^ ^]^ maize (*Z. mays*) with CRR and CRM elements;^[^ ^54^ ^]^ and goat (*C. hircus*) with numerous LTRs.^[^ ^55^ ^]^ Similarly, *L. crocea* centromeres contain dense interspersion of Cen‐42 and LTR/ERV1 elements. LTRs can form heterochromatin and contribute to genomic instability; their repression is often achieved through DNA methylation or histone modifications. Consistently, we observed elevated 5mC methylation levels in the centromeric regions, supporting the epigenetic silence of TEs in *L. crocea*. Despite rapid structural evolution, centromeres retain a conserved role in chromosome segregation, supporting the “multifunctional hypothesis of centromeres”.^[^ ^56^, ^57^ ^]^ Our analysis revealed that *L. crocea* centromeric regions exhibit low genetic diversity, suppressed recombination, and high genetic differentiation, while retaining transcriptional activity. The MYD population contains fewer protein‐coding genes in centromeric regions than DQ and negative Tajima's *D‐*values in MYD suggest recent selective sweeps or population expansion. Despite gene content divergence, both populations share functional enrichment in core pathways such as apoptosis, JAK‐STAT signaling, and ribosome biogenesis. Additionally, DQ‐specific enrichment in immune and antiviral pathways, such as NF‐κB signaling via *csnk2a2*, points to potential functional adaptation. However, further experimental validation is required to confirm these functions in *L. crocea*.

Despite the distinct demographic trajectories of the two populations, both experienced a sharp reduction in effective population size ≈13 years ago. This temporal concordance is unlikely to be coincidental and may reflect the influence of shared historical events or sociocultural events that broadly impacted multiple populations. One plausible explanation is the adoption and widespread implementation of genetic selection in breeding programs during that period, which may have imposed strong selective pressures, resulting in a rapid loss of genetic diversity. This pattern merits further investigation to elucidate its underlying causes and broader implications.

5S rRNA genes, essential for ribosome biogenesis.^[^ ^58^ ^]^ Unlike most species, the large yellow croaker exhibits an unusual distribution pattern: the 5S rRNA genes are dispersed across more than 10 chromosomes, forming ultra‐large tandem repeat clusters co‐localized with LINE/L2 elements. This pattern deviates from the 1–3 loci typically observed in eukaryotes,^[^ ^58^, ^59^, ^60^, ^61^, ^62^ ^]^ suggesting a species‐specific genomic rearrangement event. Similar co‐localization of TEs with 5S rRNA has been reported in other taxa, such as Asteraceae (Cassandras‐5S rRNA promoter co‐evolution^[^ ^63^ ^]^) and zebrafish (LTR‐mediated copy number regulation^[^ ^64^ ^]^). Although LINE/L2 elements are found in 5S‐rich regions of *L. crocea*, they are not exclusive to these loci. Whether they mediate 5S rRNA evolution or recombination in this species remains to be determined. Future integrative analyses combining genomics, epigenetics, and transcriptomics will be essential to uncover the regulatory mechanisms governing 5S rRNA dynamics.

Geographically, the large yellow croaker is divided into three populations. The MYD population inhabits the warm, shallow (40–60 m) waters from southern Zhejiang to the eastern Pearl River Estuary, characterized by high temperatures, strong sunlight, complex ecosystems, and significant pathogen pressure.^[^ ^65^ ^]^ High‐temperature environments often host greater pathogen abundance and virulence.^[^ ^66^, ^67^, ^68^ ^]^ In addition, high aquaculture density in these regions may further exacerbate pathogen load and transmission.^[^ ^69^ ^]^ The long‐term exposure to a diverse and intense pathogen environment may have driven the expansion of immune‐related gene families in the MYD population, reflecting possible adaptive responses to sustained immunological challenges. In contrast, the DQ population occupies deeper (60–100 m), colder, and more turbid waters from the southern Yellow Sea to the central East China Sea, influenced by riverine inputs and lower oxygen levels.^[^ ^70^ ^]^ Temperature is a well‐established factor shaping fish population structure^[^ ^71^ ^]^ while cold adaptation is thought to evolve more readily than heat tolerance.^[^ ^72^, ^73^ ^]^ Prior studies suggest that low‐temperature adaptation, rather than heat stress, may have played a more significant role in shaping genetic divergence in *L. crocea*.^[^ ^5^ ^]^ In addition, individuals of the DQ and MYD have significant differences in fatty acid composition, which is manifested as the FA ratios of [C18:1/(C20:1 + C22:1)] < 5.0 and/or C20:5/C20:4 > 4.0 were classified to DQ.^[^ ^74^ ^]^ Consistent with this, our findings indicate that the DQ population has undergone expansion of gene families related to glycolysis/gluconeogenesis, fructose metabolism, and carbohydrate digestion, while exhibiting contraction in fatty acid metabolism genes. Transcriptome analysis supports this, showing downregulation of genes associated with metabolic efficiency, consistent with cold‐environment adaptation. We propose that the DQ population has adapted to low‐temperature conditions via enhanced anaerobic metabolism, reduced fatty acid metabolic activity, and downregulation of energy‐efficient gene expression. In addition to metabolic traits, differences were also observed in pathways related to chemosensation, retinol metabolism, and circadian rhythm. The expansion of genes such as *tas1r1* in DQ may enhance chemosensory perception under low‐visibility conditions, potentially aiding foraging and reproduction. However, these hypotheses require further functional validation.

Despite the advances made possible by T2T genomes, several limitations remain. The lack of comparative genomic data for other Sciaenidae species restricts the reconstruction of centromere evolution. The absence of genome data for the NZ population also limits understanding of adaptive traits across the species’ full distribution range. Moreover, the lack of a species‐specific transposon database and functional validation system hampers the interpretation of TE functions. Future work should prioritize the development of comprehensive genomic resources for Sciaenidae, which will deepen our understanding of adaptive evolution and ecological diversification in this important fish family.

## 4 Conclusion

This study presents the first telomere‐to‐telomere (T2T) genome assemblies of the Min‐Yuedong (MYD) and Daiqu (DQ) populations of large yellow croaker (Larimichthys crocea), providing a comprehensive genomic resource that resolves centromeric, telomeric, and 5S rRNA regions with unprecedented accuracy. These assemblies significantly enhance our understanding of the structural and functional genomic landscape of this economically important species. Comparative genomic and transcriptomic analyses revealed distinct adaptations in the MYD and DQ populations driven by their unique environmental conditions.

## 5 Experimental Section

### Sample Collecting and Processing

An XY male individual and an XX female individual were sampled from Guanjingyang Aquaculture Co., Ltd (Ningde City, Fujian Province, China) and another from the Ningbo Academy of Oceanology and Fishery (Zhejiang Province, China) in June 2022. Following dissection on ice, muscle tissues were immediately frozen in liquid nitrogen and stored at −80 °C for subsequent DNA extraction. Sixteen tissues, including muscle, fin, skin, gill, eye, brain, heart, liver, spleen, intestine, stomach, swim bladder, gonad, kidney, cephalic kidney, and pyloric cecum, were frozen with liquid nitrogen at −80 °C for RNA extraction.

### DNA, RNA Isolation and Genome Sequencing

All DNA and RNA extraction, as well as library construction and sequencing using PacBio, ultra‐long ONT, Hi‐C, and Illumina platforms, were performed at Novogene (Beijing, China). The animal study protocol was approved by the Ethics Committee of Jimei University (protocol code 2021[5], approved on January 22, 2021).

### Genome de Novo Assembly

The draft assembly was conducted using HifiASM (v0.19.8)^[^ ^31^ ^]^ including the use of –ul to join ultra‐long ONT data and –h1/–h2 to join HiC reads (hifiasm ‐o lyc –h1 hic_R1.fq.gz –h2 hic_R2.fq.gz –ul ont.fq.gz pacbio_hifi.fq.gz), resulting in the primary, hap1, and hap2 assemblies. Based on the primary version, Juicer (v1.9.9)^[^ ^75^ ^]^ was used to construct the restriction site file using DpnII enzyme cutting sites (juicer.sh ‐g lyc ‐d./ ‐s DpnII ‐z./reference/lyc.p_ctg.fasta ‐y./reference/genome_DpnII.txt ‐p./reference/genome.chrom.size ‐D juicer), and run 3D‐DNA (v180922)^[^ ^32^ ^]^ with the ‐r 2 setting (run‐asm‐pipeline.sh ‐r 2./reference/lyc.p_ctg.fasta./aligned/merged_nodups.txt). The assembly was then imported into Juicebox (v1.11.08)^[^ ^76^ ^]^ for visualization and manual adjustments, resulting in the FINAL assembly. To fill gaps and incomplete telomeric regions in the assembly, Verkko (v1.4.1)^[^ ^33^ ^]^ was used to construct a complete diploid genome, with –hifi, –nano, –hic1, –hic2 (verkko ‐d./ –hifi pacbio_hifi.fq.gz –nano ont.fq.gz –hic1 hic_R1.fq.gz –hic2 hic_R2.fq.gz). This was then aligned to the FINAL genome using MUMmer4 (v4.0.0)^[^ ^34^ ^]^ with filtering parameters set to ‐i 98 and ‐l 100 000, addressing the gaps and telomeric regions. Any remaining small gaps in the genome were filled using TGS‐GapCloser (v1.0.1)^[^ ^77^ ^]^ with ONT reads (tgsgapcloser –scaff lyc_unfill.fasta.–reads ont.fq.gz –output lyc_tgsfill.fasta –pilon pilon –ngs illumina.fq.gz –samtools samtools –java java) and quarTeT (v1.16)^[^ ^78^ ^]^ with “GapFiller” based on ONT reads (python3 quartet.py GapFiller ‐d lyc_tgsfill.fasta ‐g ont.fq.gz –minimapoption ∖″‐x map‐ont∖″). The gap‐free genome was achieved through error correction and polishing with NextPolish2 (v1.4.1)^[^ ^79^ ^]^ utilizing both Illumina (sgs_options = ‐max_depth 100 ‐bwa) and HiFi data (hifi_options = ‐min_read_len 1k ‐max_read_len 150k, hifi_minimap2_options = ‐x asm20).

For telomere analysis, Python scripts and quarTeT (v1.16)^[^ ^78^ ^]^ were used to assess the telomere assembly status of each chromosome in the gap‐free genome. Chromosomes were considered to reach T2T assembly level if at least 10 telomeric repeat motifs, “TTAGGG” or “CCCTAA” were present at both chromosome ends. Chromosomes meeting this criterion were retained for further analysis. For chromosomes lacking detectable telomeric sequences, contigs from two draft assemblies were examined for telomeric repeat motifs. And aligned the contigs from the two draft assemblies to the gap‐free genome using MUMmer (v4.0.0).^[^ ^34^ ^]^ If telomeric sequences were identified in the contigs, the corresponding telomeric regions the authors incorporated into the gap‐free genome based on the alignment results. Additionally, contigs containing telomeric sequences but not yet anchored to chromosomes in the gap‐free genome were mapped back to the chromosomes to fill the missing telomeric region. As a result, a complete genome with all chromosomes achieving the T2T assembly level was obtained.

### Assessment of Genome Assemblies

Genome quality was assessed using several approaches. Illumina reads were aligned to the genomes using BWA (v0.7.17)^[^ ^80^ ^]^ (bwa mem ‐Y ‐t 2 ‐R “@RG∖tID:${sample}_${PBS_ARRAYID}∖tSM:${sample}∖tLB:$library∖tPL:illumina” lyc_T2T.fasta illumina_R1.fq.gz illumina_R2.fq.gz | sambamba markdup ‐r – mkdup.bam), PacBio HiFi and ultra‐long ONT reads were aligned using Minimap2 (v2.26)^[^ ^81^ ^]^ to calculate coverage (minimap2 ‐ax map‐ont/map‐hifi lyc_T2T.fasta ont.fq.gz/pacbio_hifi.fq.gz | samtools view ‐bSu ‐ |samtools sort ‐o map.bam | samtools depth maped.bam). The genome quality value (QV) was estimated from Illumina data using Merqury (v1.3).^[^ ^35^ ^]^ Completeness was evaluated using Compleasm (v0.2.6)^[^ ^37^ ^]^ with –autolineage and BUSCO (v5.7.1)^[^ ^36^ ^]^ with the actinopterygii_odb10 database. While genome continuity for the T2T assembly was assessed using the GCI (v1.0)^[^ ^38^ ^]^ based on HiFi and ONT reads (python GCI.py ‐r lyc_T2T.fasta –hifi pacbio_hifi.fq.gz –nano ont.fq.gz ‐t 32 ‐o lyc_T2T ‐p ‐f). Synteny analysis was performed using nucleotide alignments generated by MUMmer (v4.0.0),^[^ ^34^ ^]^ with filtering parameters set to ‐i 94 ‐l 10 000. After removing low‐quality alignments, the results were visualized using mummerplot with ‐large.

### Genome Annotation

Repetitive sequences and gene annotation were conducted for the T2T genomes of two geographically distinct populations. The repetitive sequence annotation was performed using a combination of methods. First, RepeatMasker (v4.1.4) was utilized to align the sequences against the RepBase database, specifically targeting the Sciaenidae family with default parameters (RepeatMasker ‐pa 4 ‐e ncbi ‐species “Actinopterygii” ‐dir./ ‐gff lyc_T2T.fasta). Next, RepeatModeler (v2.0.1)^[^ ^82^ ^]^ was employed to construct and predict a custom repeat library for each genome (RepeatModeler ‐engine ncbi ‐pa 4 ‐database lyc_T2T.fasta). The results from both approaches were integrated to generate comprehensive repetitive sequence annotations for the two populations.

Gene annotation encompassed both structural and functional aspects. Structural annotation was achieved through an integrative approach combining ab initio prediction, homology‐based annotation, and transcriptome‐based annotation. Iso‐Seq data were processed using lima(v2.9.0; GitHub ‐ PacificBiosciences/pbbioconda: PacBio) remove primers, “refine” of IsoSeq3 (v3.8.2)^[^ ^83^ ^]^ remove polyA and polymers of artificial primers (isoseq3 refine –require‐polya isoseq3.bam primers.fasta isoseq3.flnc.bam). “cluster” to extract full‐length transcripts from Iso‐seq (isoseq3 cluster isoseq3.flnc.bam isoseq3.polished.bam –verbose –use‐qvs) maped reference using “align” of pbmm2 (v1.13.1)^[^ ^81^ ^]^ with –sort. Illumina RNA‐seq transcripts were assembled using the HISAT2 (v2.2.1)^[^ ^84^ ^]^ + Cufflinks (v2.2.1)^[^ ^85^ ^]^ + StringTie (v2.1.4)^[^ ^86^ ^]^ pipeline. PASA (v2.5.3)^[^ ^87^ ^]^ was used to align these transcripts to the T2T genome via GMAP^[^ ^88^ ^]^ (Launch_PASA_pipeline.pl ‐c alignAssembly.config ‐R ‐g lyc_T2T.fasta ‐t transcripts.fasta.clean ‐T ‐u transcripts.fasta –ALIGNERS gmap, blat –CPU 8 –stringent_alignment_overlap 30.0 –TDN tdn.accs –MAX_INTRON_LENGTH 20 000 –TRANSDECODER), and TransDecoder (v5.5.0;https://github.com/TransDecoder/TransDecoder) was applied to generate the initial gff file (TransDecoder.Predict ‐t transcripts.fasta –retain_blastp_hits blastp.out). AUGUSTUS (v3.5.0)^[^ ^89^ ^]^ and SNAP (v2.61.2)^[^ ^90^ ^]^ were used for model training and ab initio prediction. Homology‐based annotation involved aligning the target genome with those of nine closely related species, including *L. crocea*, *D. labrax*, *G. aculeatus*, *T. rubripes*, *G. morhua*, *O. latipes*, *D. rerio*, *N. albiflora*, *C. lucidus*, and *L. polyactis*, using Exonerate (v2.4.0)^[^ ^91^ ^]^ to predict exons.

The final gff file was generated by integrating all evidence using EVM (v2.1.0)^[^ ^92^ ^]^ with ABINITIO_PREDICTION 2 TRANSCRIPT 8 PROTEIN 4. Functional annotation was conducted using multiple databases, including Pfam, Swiss‐Prot, TrEMBL, GO, KOG/COG, and KEGG. For non‐coding RNAs (ncRNAs), rRNAs, snoRNAs, and snRNAs were identified using CMSCAN.^[^ ^93^ ^]^ tRNA structures were predicted using tRNAscan‐SE (v2.0.12).^[^ ^94^ ^]^ Transposable elements were classified and identified using LTR_finder^[^ ^95^ ^]^ and LTR_retriever (v3.0.1).^[^ ^96^ ^]^

Previously published genome versions (MYD: GCA_000 972 845.2, GCA_0 043 52675.2, GCA_04 018 2605.1. DQ: GCA_04 018 2835.1) were compared with the T2T genome using Liftoff (v1.6.3)^[^ ^97^ ^]^ to evaluate improvements in genome assembly and annotation.

The identification of 5S rRNA was performed using RepeatMasker (v4.1.4, http://www.repeatmasker.org) with ‐e ncbi, ‐species “Actinopterygii” for the statistics and identification of repeated sequences, and awk was used to count the number of identified copies and locate them.

### Identification of Telomere and Centromere

High‐quality genome assemblies offer a valuable opportunity to elucidate centromeric regions. To pinpoint centromeric satellite sequences, the most abundant repetitive elements were extracted from the genome assembly using RepeatMasker (v4.1.4) analysis. Furthermore, centromeric regions were delineated through the application of Centromics (v0.3; GitHub ‐ zhangrengang/Centromics: Centromics: visualing centromeres with multiple omics data) based on ONT and HiC data (centromics ‐l ont.fq.gz ‐g lyc_T2T.fa ‐pre onthic ‐hic hic.merged.txt.gz ‐outdir onthic), and quarTeT (v1.16)^[^ ^78^ ^]^ with CentroMiner (python3 quartet.py CentroMiner ‐i lyc_T2T.fasta ‐p lyc_T2T.cen). Telomeric sequences were characterized by identifying tandem repeats of the motif “TTAGGG / CCCTAA” using quarTeT (v1.16)^[^ ^78^ ^]^ with TeloExplorer (python3 quartet.py TeloExplorer ‐i lyc_T2T.fasta ‐c animal ‐p lyc_T2T.telo).

The Cen‐42 sequence was compared and analyzed in *L. polyactis, N. albiflora*, and *C. lucidus* using BLASTN. Among them, the genome for *N. albiflora* was obtained from the GenBank (accession no. GCA_902 410 095.1^[^ ^44^ ^]^), while the genome for *L. polyactis* and *C. lucidus* was determined by the laboratory and was not publicly published yet.

### Gene Family and Phylogenomic Analysis

The T2T genomes of the two L. crocea, along with 11 homologous species (Argyrosomus japonicus, C. lucidus, Danio rerio, Dicentrarchus labrax, Gadus morhua, Gasterosteus aculeatus, N. albiflora, Nibea coibor, Oryzias latipes, Protonibea diacanthus, and Takifugu rubripes) and two outgroup species (Homo sapiens and Mus musculus), were used for gene family clustering and phylogenetic analysis. The longest transcript from each species was selected, and genes encoding proteins shorter than 30 amino acids or containing internal stop codons were excluded. Orthologous genes were identified using OrthoFinder (v2.5.5),^[^ ^98^ ^]^ and gene trees were inferred using the ‐M msa option. The species tree was constructed with IQ‐TREE (orthofinder ‐f./ ‐d ‐M msa ‐S diamond ‐T iqtree ‐t 48 ‐a 48). The resulting trees were then processed in R8S (v1.81)^[^ ^99^ ^]^ to generate an ultrametric tree, and CAFE5 (v5.1)^[^ ^100^ ^]^ with ‐*p* 0.05 was employed to analyze and visualize gene family contraction and expansion. The visualization of the phylogenetic tree was performed using tvBOT.^[^ ^101^ ^]^ Gene families that exhibited significant contraction or expansion were subjected to KEGG enrichment analyses to elucidate their potential biological functions.

Selective pressure analysis between the DQ and MYD were compared using WGDI (v0.6.5),^[^ ^102^ ^]^ with collinearity *e*‐value = 1*e *− 5, grading = 50, 40, and 25 mg = 40, 40, *P*‐value = 0.2, blockinfo *e*‐value = 1*e *− 5, repeat_number = 20. Gene information was extracted for collinearity analysis and Ka/Ks (Non‐synonymous/synonymous substitution rate) calculations.

### SV Analysis Based on Long Reads

The SVs analysis of the two *L. crocea* populations was conducted using Pacbio HiFi data and high‐depth Illumina data. Initially, HiFi reads and genomes from MYD (MYD_XX, MYD_XY, SRR29302600) and DQ (DQ_YY, SRR29302596) individuals were aligned to the T2T‐DQ using CuteSV (v2.1.1),^[^ ^103^ ^]^ pbsv (v2.10.0; GitHub ‐ PacificBiosciences/pbsv) with –call‐min‐reads‐one‐sample 50, –call‐min‐reads‐all‐samples 50 (pbsv call ‐O 5 ‐j 30 lyc_T2T.fasta lyc.svsig.gz lyc.pbsv.vcf –call‐min‐reads‐one‐sample 50 –call‐min‐reads‐all‐samples 50), and SyRI (v1.6.3)^[^ ^104^ ^]^ to identify SVs (python3 syri ‐c map.bam ‐r lyc_DQ_T2T.fasta ‐q querygenome ‐k ‐F B). The SVs detected by the three programs were combined using the “merge” function (SURVIVOR merge merge_files 1000 2 1 1 0 30 merged.vcf) in SURVIVOR (v 1.0.7).^[^ ^105^ ^]^

### Genetic Diversity Analysis

The whole genome resequencing data from 20 MYD individuals and 21 DQ individuals were used for analysis. Clean reads were first aligned to the reference genome using BWA (v0.7.17)^[^ ^80^ ^]^ to generate BAM files. Two parallel pipelines were then employed: 1) SVs were identified using “call” function (delly call ‐g lyc_T2T.fasta ‐o delly.bcf samples.bam) of Delly (v1.2.6),^[^ ^106^ ^]^ and population‐specific SVs were further screened through comparative analysis, Combined with the analysis results of long reads, the variations that existed in MYD but not in DQ are selected as MYD‐specific variations. On this basis, the internal script was used to filter the variations with completely consistent genotypes in MYD; 2) SNP calling was performed using the GATK (v4.1.9.0)^[^ ^107^ ^]^ pipeline to generate VCF files: GVCF file using HaplotypeCaller with –pcr‐indel‐model CONSERVATIVE, –read‐filter MappingQualityReadFilter –minimum‐mapping‐quality 20. SNP filter using VariantFiltration with –filter‐expression “QD < 2 || MQ < 40 || FS > 60 || SOR > 3 || MQRankSum < −12.5 || ReadPosRankSum < −8”, and bcftools(v1.19)^[^ ^108^ ^]^ filter with ‐i 'FILTER = “PASS” and F_MISSING < 0.2′. Finally, VCFtools^[^ ^109^ ^]^ was used to filter out SNPS with –maf 0.05 and –hwe 0.00001. Followed by quality filtering to ensure high‐confidence variant sites. Finally, population genetic statistics, including nucleotide diversity (*π*), Tajima's D, and FST, were calculated in 10 kb sliding windows using VCFtools.^[^ ^109^ ^]^ Recombination rates were estimated using fastEPRR.^[^ ^110^ ^]^ Effective population size (Ne) was inferred using PSMC (v0.6.5)^[^ ^39^ ^]^ with the parameters ‐p “4 + 25 × 2 + 4 + 6”, ‐t 2, and ‐r 5. The results were visualized using the psmc_plot.pl script with ‐g 2 ‐u 0.5 × 10^−8^. To generate the consensus sequence required for the PSMC analysis in the specific psmcfa format, the authors first used the following pipeline based on samtools, bcftools, and vcfutils: samtools mpileup ‐Q 30 ‐q 30 ‐u ‐v ‐f genome.fasta sample.bam | bcftools view ‐c ‐ |bcftools filter ‐e “F_MISSING > 0.2 || MAF < 0.05”| vcfutils.pl vcf2fq ‐d 15 ‐D 45 | gzip > diploid.fq.gz. The resulting sequence was then converted to psmcfa format using the fq2psmcfa, as required for PSMC input. As there is currently no available research on the population‐level mutation rate of *L. crocea*, the mutation rate estimated for its closest evolutionary relative, the three‐spined stickleback (*G. aculeatus*),^[^ ^111^ ^]^ as a proxy was employed for the calculations. ‐g 2 means the generation time of *L. crocea* is two years. GONE2(v2.0)^[^ ^112^ ^]^ was used to infer the changes in the effective population size in recent years (gone2 ‐r 3.3 mydz.vcf.tped). The value of the parameter ‐r was determined following the study by Yu et al.,^[^ ^113^ ^]^ using the average of the female (3.55) and male (3.05) values.

### Methylation and A/B Compartments Determination

Methylation analysis was performed using ONT reads. First, the ONT reads were processed for basecalling with ont‐guppy‐cpu (v3.4.1)^[^ ^114^ ^]^ based on FAST5 format (guppy_basecaller ‐i./fast5_ single.fast5/‐s./guppy‐results/basecalling ‐c ont‐guppy‐cpu/data/dna_r9.4.1_450bps_fast.cfg –fast5_out on –num_callers 4 –cpu_threads_per_caller 3 –compress_fastq). The resulting reads were then indexed and aligned to the T2T genome using Minimap2 (v2.26).^[^ ^81^ ^]^ Methylation sites featuring 5mC modifications were identified using Nanopolish (v0.14.0)^[^ ^115^ ^]^ with “call‐methylation” and filtered with “calculate_methylation_frequency.py”. Finally, methylation coverage was evaluated within 10, 100 kb windows via “intersect” of BEDTools (v2.31.0).^[^ ^116^ ^]^

Hi‐C data were processed using HiC‐Pro (v3.1.0)^[^ ^117^ ^]^ to generate contact maps at resolutions of 100, 200, 250, 300, and 500 kb, LIGATION_SITE  =  GATC, MATRIX_FORMAT  =  complete. A/B compartments were identified using FANC (v0.9.27)^[^ ^118^ ^]^ based on a 250 kb ICE‐normalized contact matrix.

### FISH Experiment

Mid‐metaphase chromosomes were prepared from *L. crocea* macrophages, and FISH experiments were conducted following the protocol described by Huang et al.^[^ ^23^ ^]^ Probes specific to the identified centromeric and telomeric DNA sequences were labeled with biotin‐16‐dUTP and digoxigenin‐11‐dUTP via nick translation using reagents from Roche Diagnostics (https://www.roche.com/about/business/diagnostics.htm). Post‐hybridization signals for biotin‐labeled probes were detected using Alexa Fluor 488‐conjugated streptavidin (Life Technologies, now part of Thermo Fisher Scientific, https://www.thermofisher.com), while those for digoxigenin‐labeled probes were detected using rhodamine‐conjugated anti‐digoxigenin antibodies (Roche Diagnostics). Chromosomes were counterstained with DAPI in Vectashield antifade mounting medium. Fluorescence images were captured using an Olympus BX63 fluorescence microscope (Olympus, Tokyo, Japan) and subsequently processed using Adobe Photoshop 2022 (Adobe, https://www.adobe.com).

### Transcriptome Sequencing and Analysis

Clean reads were then aligned to the T2T genome using STAR (v2.7.7),^[^ ^119^ ^]^ with –clip5pNbases 0 5, –clip3pNbases 0 25, –outSJfilterOverhangMin 30 16 16 16, –outSJfilterCountUniqueMin 4 2 2 2, –alignSJoverhangMin 6, –alignIntronMax 500 000, and aligned reads were assembled into transcripts using StringTie (v2.1.4).^[^ ^86^ ^]^ Read counts were extracted using FeatureCounts (v2.0.5)^[^ ^120^ ^]^ with ‐s 0, ‐p, ‐Q 20, –fracOverlap 0.8. Differential expression analysis was performed using DESeq2^[^ ^121^ ^]^ and genes with |log2FoldChange| > 1 and adjusted *P*‐value (*P*adj) ≤ 0.05 were considered differentially expressed.

### Statistical Analysis

All data were analyzed using SPSS 20.0 software (IBM, USA). Differences were considered statistically significant at *P* < 0.05, as determined by one‐way ANOVA with LSD multiple comparisons.

## Conflict of Interest

The authors declare no conflict of interest.

## Author Contributions

Y.C. dealt with conceptualization, methodology, bioinformatics work and writing the original draft. Y.Y., B.W., and W.S. dealt with methodology and bioinformatics work. X.W., F.H., and B.W. dealt with sample collection, managed the field work, and performed the experiments. M.C. and M.F. conceived and wrote the review. Z.W. is the corresponding author. Z.W. conceived, supervised, wrote the review and editing, and dealt with the funding acquisition. All authors read and approved the manuscript.

## Supporting information



Supporting Information

Supporting Information

Supporting Information

Supporting Information

Supporting Information

## Data Availability

All the PacBio long DNA reads, Hi‐C reads, ONT reads, Illumina short RNA reads and DNA reads, and PacBio long RNA reads are available from NCBI via the accession numbers PRJNA1247177, PRJNA1247232, PRJNA1247692, PRJNA1247709, PRJNA1215328. The assembled genomes of MYD and DQ have been deposited at GenBank under JBMWUI000000000 and JBMWUJ000000000, respectively. The accession number will be available upon acceptance or publication.
