# Supplementary figures and images for: T2T Genomes Unveil Centromere Architecture and Adaptive Divergence in Large Yellow Croaker (Larimichthys crocea)
Source: Adv Sci (Weinh). 2025 Aug 22;12(43):e06374. doi: 10.1002/advs.202506374 (PMC12631908; doi:10.1002/advs.202506374)

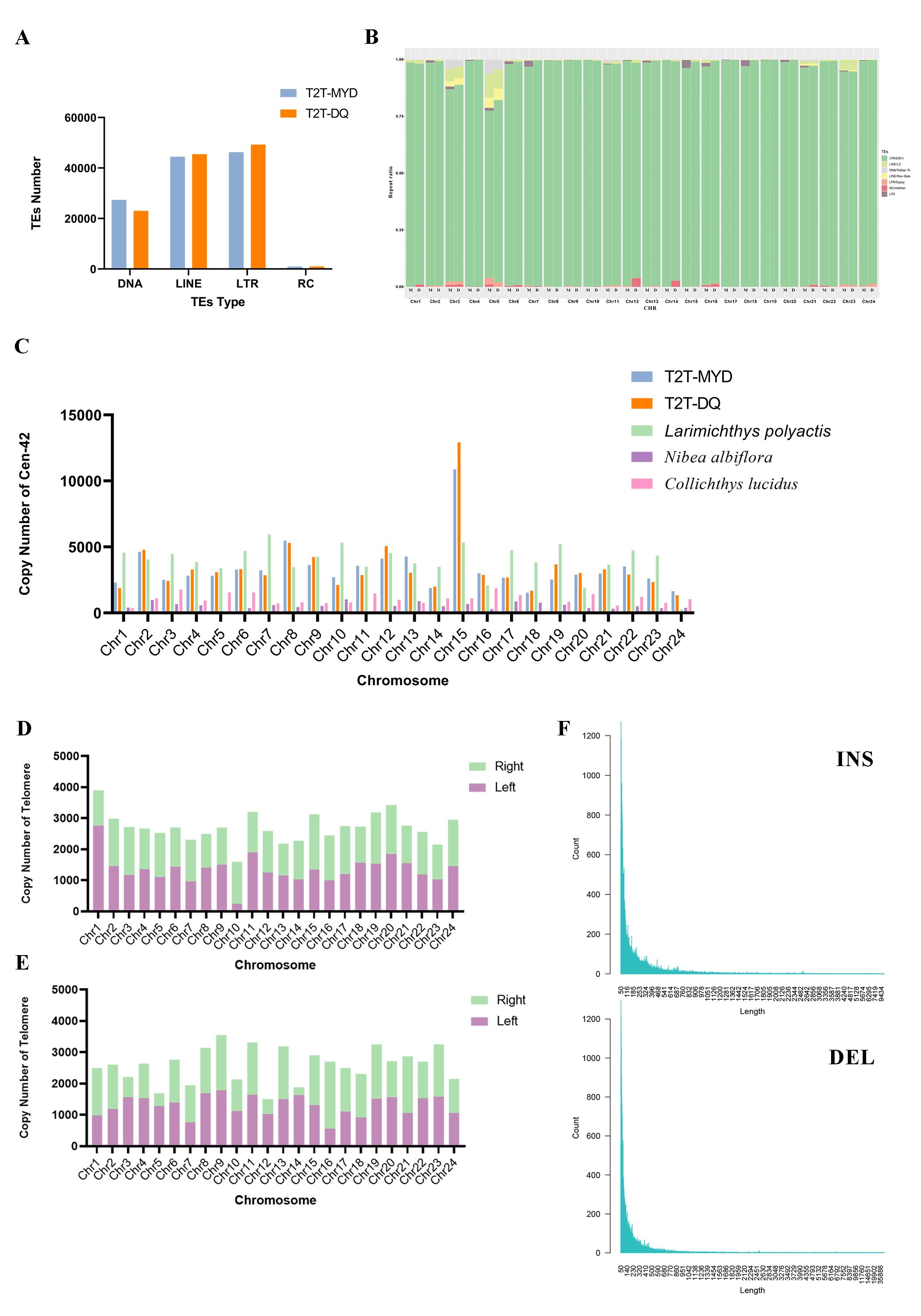

Supplement: Supplementary file 1 — Supporting Information [file ADVS-12-e06374-s001.tif]

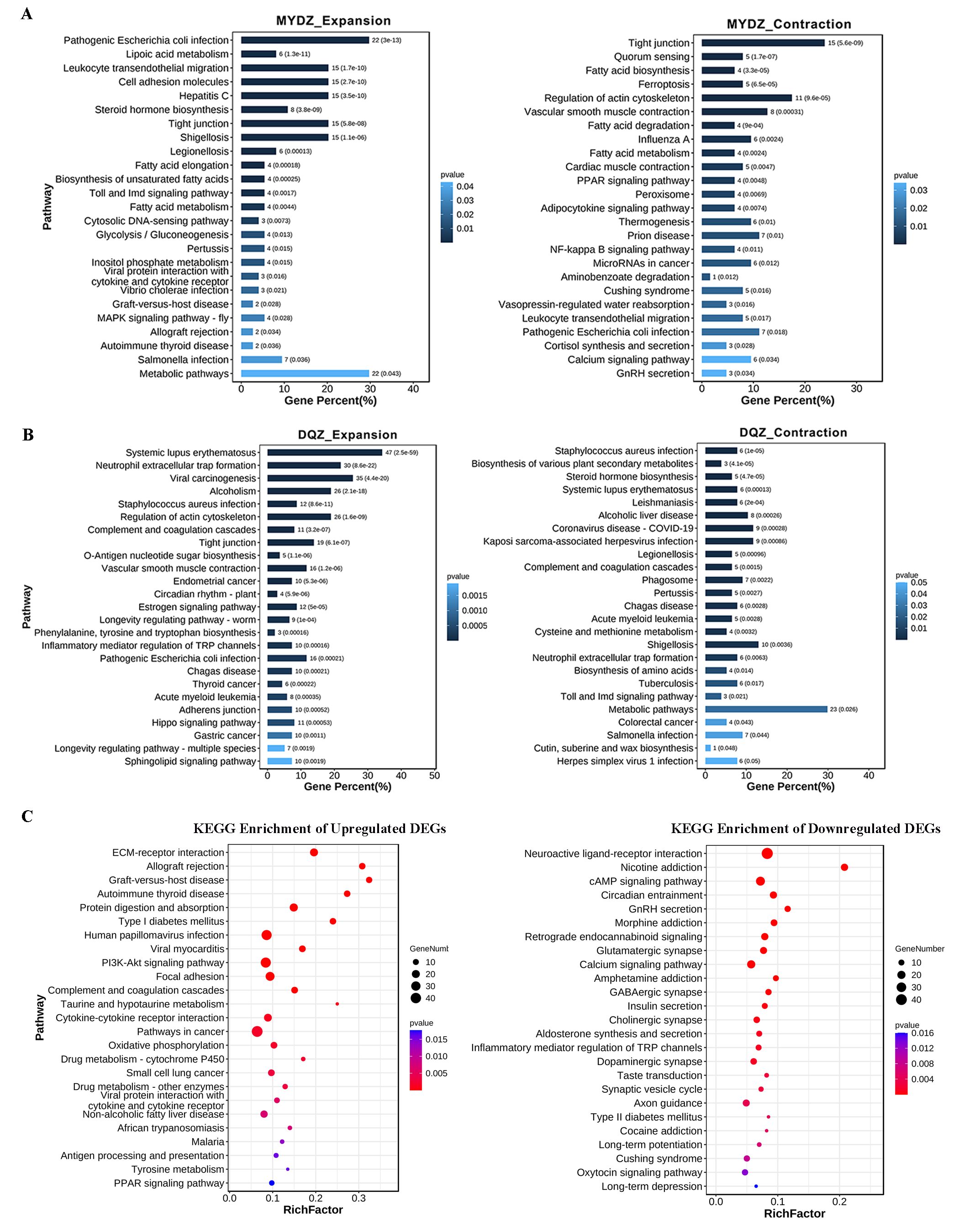

Supplement: Supplementary file 2 — Supporting Information [file ADVS-12-e06374-s005.tif]
