# Supplementary material for: T2T Genomes Unveil Centromere Architecture and Adaptive Divergence in Large Yellow Croaker (Larimichthys crocea)
Source: Adv Sci (Weinh). 2025 Aug 22;12(43):e06374. doi: 10.1002/advs.202506374 (PMC12631908; doi:10.1002/advs.202506374)
Supplement: Supplementary file 5 — Supporting Information [file ADVS-12-e06374-s003.docx]

Figure S1

A) Quantification of four types of transposable elements in the genomes of two populations. Blue represents the T2T-MYD, and orange represents the T2T-DQ. B) Classification and relative abundance of transposable elements in centromeric regions of T2T-MYD (left) and T2T-DQ (right) genomes. Green denotes LTR/ERV1 elements. C) Copy numbers of the Cen-42 sequence across T2T-MYD, T2T-DQ, and genomes of *Larimichthys polyactis*, *Nibea albiflora* and *Collichthys lucidus*. D) Copy number of the telomeric repeat motif “TTAGGG” at both ends of each chromosome for T2T-MYD. Green means right ends and purple means left ends. E) Copy number of the telomeric repeat motif “TTAGGG” at both ends of each chromosome for T2T-DQ. Green means right ends and purple means left ends. F) Insertion (INS) and deletion (DEL) variant length distributions between two populations.

Figure S2

A) KEGG functional enrichment of expanded and contracted gene families in T2T-MYD. B) KEGG functional enrichment of expanded and contracted gene families in T2T-DQ. C) KEGG pathway enrichment analysis of DEGs.

Table S1

Summary of assemblies for two populations of Large Yellow Croaker

Table S2

Summary of Sequencing Data for Two Populations of Large Yellow Croaker

Table S3

Sequencing Metrics for T2T-MYD and T2T-DQ

Table S4

Evaluation of Genome Assembly and Annotation Results

Table S5

Statistics for the Two Populations Count

Table S6

Structural and Functional Annotation Results of Genomes

Table S7

ncRNA Count of Genomes

Table S8

Comparison of Additional Genes Annotated in the T2T Genome Assembly Compared to Other Genome Versions

Table S9

Copy Number of Telomere

Table S10

The top5 repeats with copy numbers of Large Yellow Croaker

Table S11

Centromere Characteristics and Chromosome Type Identification in two genomes

Table S12

The copy numbers of Cen-42 in Sciaenidae

Table S13

Statistics of gene activity in the centromere region

Table S14

Homology Analysis of Centromeric Region Genes

Table S15

KEGG Enrichment Results

Table S16

5S rRNA Element Distribution Across Chromosomes

Table S17

The Copy Number of LINE/L2 in the region harboring 5S rRNA genes

Table S18

Summary of Gene Family Clustering

Table S19

Summary of Evolutionary Characteristics and Expression Patterns of Functional Genes in Large Yellow Crosker

Table S20

Summary of SVs in Functional Genes using HiFi Reads

Table S21

Summary of SVs in Functional Genes for MYD

Table S22

DEGs of Brains from DQ and MYD populations

Table S23

The effective population size prediction uses GONE2 for DQ and MYD populations

Data S1

Newly Annotated Genes in T2T Genomes Compared to Other Versions

Data S2

Genomic Feature Statistics in 100 kb Windows for T2T-MYD

Data S3

Genomic Feature Statistics in 100 kb Windows for T2T-DQ

Data S4

KEGG Enrichment Analysis of Protein-Coding Genes in T2T-MYD Centromeric Regions

Data S5

KEGG Enrichment Analysis of Protein-Coding Genes in T2T-DQ Centromeric Regions

Data S6

KEGG Enrichment Analysis of Expanded Genes in T2T-MYD

Data S7

KEGG Enrichment Analysis of Contracted Genes in T2T-MYD

Data S8

KEGG Enrichment Analysis of Expanded Genes in T2T-DQ

Data S9

KEGG Enrichment Analysis of Contracted Genes in T2T-DQ

Data S10

Information of Collinearity Blocks in T2T-DQ and T2T-MYD

Data S11

Ka/Ks Ratios for Gene Pairs Between T2T-DQ and T2T-MYD

Data S12

KEGG Enrichment Analysis of Positively Selected Genes

Data S13

Transcriptome Differential Expression Analysis Results

Data S14

KEGG Enrichment Analysis of Upregulated DEGs

Data S15

KEGG Enrichment Analysis of Downregulated DEGs
